# CTC-177, a novel drug–Fc conjugate, shows promise as an immunoprophylactic agent against multidrug-resistant Gram-negative bacterial infections

**DOI:** 10.1093/jacamr/dlae100

**Published:** 2024-07-26

**Authors:** Arianne Lovey, Annie Lee, Allison Yu, Mila Krel, Mingming Wang, Padmaja Paderu, Thomas Brady, Grayson Hough, Qiping Zhao, James M Balkovec, David S Perlin, Yanan Zhao

**Affiliations:** Center for Discovery and Innovation, Hackensack Meridian Health, Nutley, NJ 07110, USA; Center for Discovery and Innovation, Hackensack Meridian Health, Nutley, NJ 07110, USA; Center for Discovery and Innovation, Hackensack Meridian Health, Nutley, NJ 07110, USA; Center for Discovery and Innovation, Hackensack Meridian Health, Nutley, NJ 07110, USA; Center for Discovery and Innovation, Hackensack Meridian Health, Nutley, NJ 07110, USA; Center for Discovery and Innovation, Hackensack Meridian Health, Nutley, NJ 07110, USA; Departments of Medicinal Chemistry and Protein Chemistry, Cidara Therapeutics, Inc., San Diego, CA 92121, USA; Departments of Medicinal Chemistry and Protein Chemistry, Cidara Therapeutics, Inc., San Diego, CA 92121, USA; Departments of Medicinal Chemistry and Protein Chemistry, Cidara Therapeutics, Inc., San Diego, CA 92121, USA; Departments of Medicinal Chemistry and Protein Chemistry, Cidara Therapeutics, Inc., San Diego, CA 92121, USA; Center for Discovery and Innovation, Hackensack Meridian Health, Nutley, NJ 07110, USA; Hackensack Meridian School of Medicine, Hackensack Meridian Health, Nutley, NJ 07110, USA; Lombardi Comprehensive Cancer Center, Georgetown University, Washington, DC 20057, USA; Center for Discovery and Innovation, Hackensack Meridian Health, Nutley, NJ 07110, USA; Hackensack Meridian School of Medicine, Hackensack Meridian Health, Nutley, NJ 07110, USA

## Abstract

**Background:**

The widespread emergence of antibiotic resistance including MDR in Gram-negative bacterial pathogens poses a critical challenge to the current antimicrobial armamentarium.

**Objectives:**

To create a novel drug–Fc conjugate (DFC) that can be delivered at sustained and prolonged levels while simultaneously activating the host immune response to combat MDR Gram-negative infections.

**Methods:**

The Cloudbreak™ platform was used to develop DFCs consisting of a targeting moiety (TM) (a polymyxin-derived dimer) attached via a non-cleavable linker to an effector moiety (EM) (the Fc domain of human IgG1). *In vitro* activities of the DFCs were assessed by MIC testing. Neutropenic mouse models of thigh infection, septicaemia and pneumonia were used to evaluate *in vivo* efficacy. Pharmacokinetics were evaluated in mice and cynomolgus monkeys.

**Results:**

A single prophylactic dose of our lead DFC, CTC-177, resulted in significantly decreased bacterial burdens and reduced inflammation comparable to daily treatment with colistin in septicaemia and pneumonia mouse models. Furthermore, CTC-177 prophylaxis was able to restore colistin efficacy in colistin-resistant septicaemia, reducing bacterial burdens beyond the limit of detection. Finally, CTC-177 displayed a long terminal half-life of over 24 and 65 h in mice and cynomolgus monkeys, respectively.

**Conclusions:**

These data support the continued development of Cloudbreak™ DFCs as broad-spectrum prophylactic agents against Gram-negative infections.

## Introduction

It has been reported that approximately 42% of hospital-acquired infections in ICU patients are associated with Gram-negative bacteria, particularly *Acinetobacter*, *Klebsiella* or *Pseudomonas* species.^1^ Notably, successful treatment of these Gram-negative infections has become considerably more challenging in recent years, primarily due to significant increases in the pathogens resistant to existing antimicrobial agents. Following the CDC’s 2019 classification of drug-resistant *Acinetobacter baumannii*, *Klebsiella pneumoniae* and *Pseudomonas aeruginosa* as urgent or serious threats,^2^ hospital onset of carbapenem-resistant *A. baumannii* infections surged by 78% in 2020. Concurrently, carbapenem-resistant Enterobacteriaceae infections and MDR *P. aeruginosa* infections rose 35% and 32%, respectively,^3^ posing a persistent threat to immunocompromised patients.

Several critical factors have been identified to be associated with antibiotic resistance in Gram-negative pathogens, including constitutive expression of efflux systems, expression of class B β-lactamases, the ability to form biofilms, and plasmid-carried resistance mechanisms such as the *mcr-1* gene.^4–10^ The antibiotic resistance resulting from combinations of these factors cause a significant reduction in antibiotic options for clinicians^11,12^ and often result in prolonged treatment and extended hospital stays, which potentiate antibiotic resistance and spread of infection.

Many groups have worked on alternative approaches to antibiotic therapy, including the use of antimicrobial peptides, bacteriophages and vaccination strategies.^13–15^ In a recent proof-of-concept study,^16^ we demonstrated that drug–Fc conjugates (DFCs) [formerly referred to as antibody–drug conjugates (ADCs)] hold promise to provide alternative therapeutic options for drug-resistant Gram-negative infections. These DFCs are designed as bispecific molecules using the Cloudbreak^™^ platform developed at Cidara Therapeutics, Inc. (San Diego, CA, USA), to combine the antibacterial targeting moiety (TM) and the effector moiety (EM), which engages innate immune components to fulfil dual killing of the Gram-negative bacteria and bind the neonatal receptor (FcRn), leading to favourable pharmacokinetic (PK) behaviour. Specifically, these TMs are novel dimeric polymyxin molecules designed to tightly bind LPS despite colistin resistance-associated structural modifications. The EM is the Fc fragment of human IgG1 (hIgG1). The final conjugated compounds were effective at inhibiting growth of *A. baumannii* in a neutropenic thigh infection model.

In this study, we show that one such DFC molecule, CTC-177, is an effective immunoprophylactic agent in multiple animal models of infection including the most clinically relevant septicaemia and pneumonia models.^4,9,17–21^ CTC-177 prophylaxis not only resulted in significantly lower bacterial burdens and improved survival when mortality was involved, but importantly induced sensitization to colistin in the scenario of colistin-resistant infection, leading to clearance of the infection.

## Materials and methods

### CTC-177 synthesis

**Figure 1.**
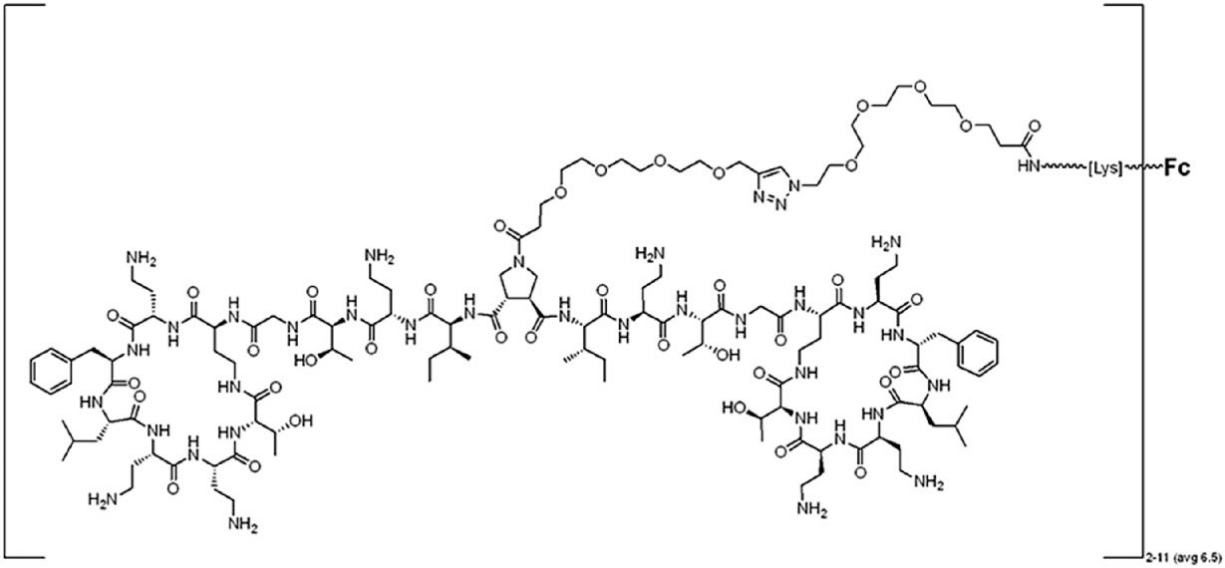
Structure of CTC-177. Novel dimeric polymyxin-targeting moiety structure containing a centrally linked PEG chain attached to hIgG1 Fc protein for an average DAR of 6.5 (2–11) (average molecular weight = 72 111 Da).

CTC-177 (Figure 1) was synthesized utilizing a previously published process^16^ in which the TM (dimeric polymyxin molecules containing a centrally linked alkynyl group) and hIgG1 Fc protein^22^ are independently synthesized. The Fc protein is modified, resulting in the azido-PEG4-Fc intermediate. Finally, the azido-PEG4-Fc [1.0 eq, drug-to-antibody ratio (DAR) ∼6.5] is conjugated with the TM (10–12 molar eq) dissolved in PBS using the Cu(I)-catalysed Huisgen 1,3-dipolar cycloaddition with several modifications (Click reaction) over a 2 h period. The synthesis of the TM for CTC-177 is described in Figure S1 (available as Supplementary data at *JAC-AMR* Online).

### Antibiotics and bacterial strains

Colistin sulphate (PHR1605-1G, Sigma) solution was prepared for administration to mice in 1× PBS. Clinical isolates of *K. pneumoniae*, *A. baumannii*, *P. aeruginosa* and *Escherichia coli* obtained from the bacterial repository in Kreiswirth lab at the Center for Discovery and Innovation (CDI) and ATCC control strains (*K. pneumoniae* ATCC 700603, *P. aeruginosa* ATCC 10145 and ATCC 27853, and *E. coli* ATCC 25922) were refreshed on LB agar and prepared in Mueller–Hinton Broth (MHB) prior to experiments. MICs were determined for TMs and ADCs using CLSI broth microdilution methodology.^23^

### Animals

Male (PK only) and female 6-week-old CD-1 mice weighing 22–26 g (Charles River Laboratory) were used for all mouse models in this study. Mice were housed in the Animal Biosafety Level-2 Research Animal Facility at CDI, Hackensack Meridian Health (HMH). All experimental procedures were performed in accordance with National Research Council guidelines and approved by the HMH Institutional Animal Care and Use Committee (IACUC). Cynomolgus monkeys were purchased and housed by Inotiv. All procedures were performed under Inotiv IACUC.

### Neutropenic murine models

Three neutropenic mouse models, deep-thigh infection, septicaemia and pneumonia, were used in our study. Neutropenia was induced utilizing 150 or 100 mg/kg cyclophosphamide via intraperitoneal (IP) injection on Day −4 and Day −1, respectively, prior to infection. Indicated strains were grown at 37°C with shaking overnight. The culture was centrifuged, washed and OD_600_ adjusted to get 1.5–2.0 × 10^8^ cfu/mL (thigh model), 1.0 × 10^9^ cfu/mL (pneumonia model) or 3.0–4.0 × 10^8^ cfu/mL (sepsis model). Sepsis inocula were adjusted to 1.0 × 10^8^ for *K. pneumoniae* infections or 2.0 × 10^7^ for *P. aeruginosa* infections. To infect the mice, the mice were manually restrained (thigh infection model) or anaesthetized using isoflurane gas (sepsis and pneumonia infection models) and infected with 0.05 mL of bacterial inocula via intramuscular injection (thigh infection model), retro-orbital injection (sepsis infection model) or intranasal instillation (pneumonia model). Following infection, mice were observed twice daily. This observation included monitoring for weight loss as a possible sign of acute toxicity. No abnormal clinical signs or morbidity were observed. At each study timepoint, mice were humanely euthanized by CO_2_ asphyxiation. Thighs (thigh infection model), kidneys (sepsis infection model) and/or lungs (sepsis and pneumonia infection models) were removed, dissociated and diluted on LB agar to enumerate bacterial burdens.

### Treatment administration

#### DFC administration

**Figure 2.**
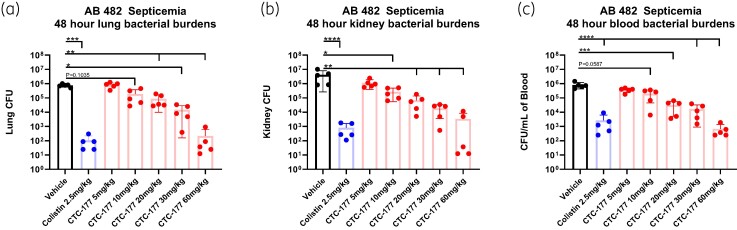
CTC-177 minimal effective dose. Single prophylactic dose of CTC-177 administered IP at 5, 10, 20, 30 or 60 mg/kg at 12 h prior to infection as indicated. Colistin (2.5 mg/kg, SC) and vehicle administered twice daily commencing 1 h post infection. Mice were sacrificed 48 h post infection; (a–b) burdens displayed as the total burdens in the lung and kidney, or (c) cfu/mL of blood at 48 h post infection. *P < 0.05; **P < 0.01; ***P < 0.001; ****P < 0.0001 (*n* = 5). AB, *A. baumannii*.

**Figure 3.**
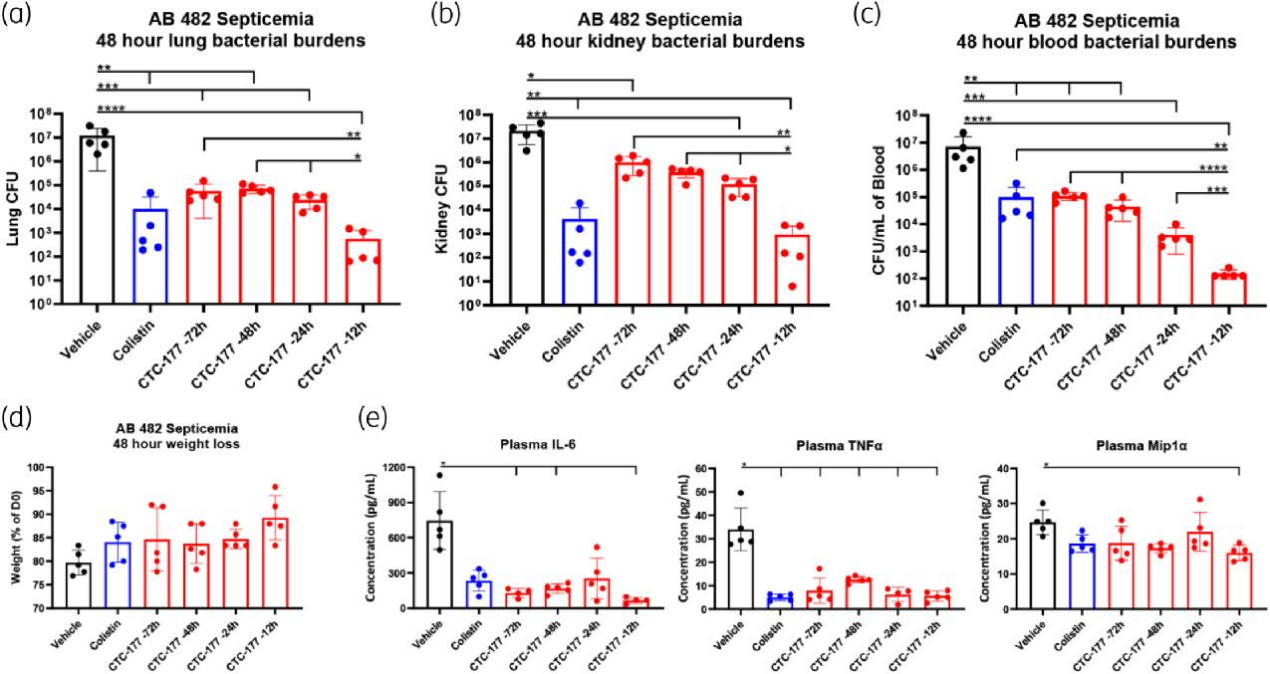
Dose–time response demonstrates that optimal bacterial control is achieved by a single immunoprophylactic dose of CTC-177 at 60 mg/kg 12 h prior to infection. Single prophylactic dose of CTC-177 administered IP at 60 mg/kg at 72, 48, 24 or 12 h prior to infection as indicated. Colistin (2.5 mg/kg, SC) and vehicle administered twice daily commencing 1 h post infection. Mice were sacrificed 48 h post infection; (a–b) burdens displayed as the total burdens in the lung and kidney, or (c) cfu/mL of blood at 48 h post infection. (d) Percentage of weight of each mouse relative to time of infection. (e) Cytokine/chemokine levels in plasma at 48 h post infection. *P < 0.05; **P < 0.01; ***P < 0.001; ****P < 0.0001 (*n* = 5). *P* values are corrected for multiple comparisons as described in the methods. AB, *A. baumannii*.

In the initial *in vivo* screen, a two-dose prophylactic regimen was used. Specifically, CTC-177 was administered intraperitoneally at 30 mg/kg (=0.42 µmol/kg) at 12 h prior to infection with a booster dose of 30 mg/kg administered 1 h following infection. In the experiment to explore the minimal effective dose of CTC-177 (Figure 2), a single dose of CTC-177 was administered IP at the indicated concentrations 12 h prior to infection. To optimize the prophylactic window (Figure 3), a single dose of CTC-177 was administered IP at 60 mg/kg (=0.83 µmol/kg) either 72, 48, 24 or 12 h prior to infection. In the experiment to test the potential of CTC-177 restoring colistin IP at 60 mg/kg 12 h prior to infection, with or without colistin treatment starting from 1 h post infection.

#### Colistin treatment

Colistin was administered subcutaneously at 2.5 mg/kg (=2.2 µmol/kg) (thigh and sepsis infection model) or 10 mg/kg (=8.7 µmol/kg) (pneumonia infection model) twice daily beginning 1 h post infection.

#### Vehicle treatment

Two hundred microlitres of PBS was administered IP twice daily beginning 1 h post infection.

### Cytokine ELISA

Lung and kidney tissue was homogenized by gentleMACS dissociation in 2.5 mL of PBS. Plasma was isolated from K_2_EDTA-treated tubes. Samples were maintained at −20°C in the presence of 2× HALT protease inhibitor until the time of ELISA. IL-6, TNFα and MIP1α concentrations were quantified using commercially available kits (88-7064-88, 88-7324-88 and 88-56013-88).

### PK

Single-dose PK studies were performed in uninfected mice. The PK parameters for CTC-177 were evaluated in male and female CD-1 mice (4–5 animals/group) after 20 mg/kg IV administration. Whole blood samples were collected via tail vein at 0.3, 1, 2, 4, 8, 24 h, or cardiac puncture at 48 h post injection. A single-dose PK study was also performed in two healthy cynomolgus monkeys (1 male and 1 female) (Inotiv). For monkeys, blood was collected at 1, 4, 8, 24, 48, 72, 120, 168, 240 and 336 h post injection. Plasma DFC concentrations at each timepoint were measured by ELISA, as detailed below. PK parameters were analysed by non-compartmental analysis using Phoenix WinNonlin software.

### PK ELISA

For mouse PK, TM ELISAs were completed utilizing the Takara Peptide Coating Kit (MK100, Takara Bio) and LPS from *E. coli* O127:B8 (L4516, Sigma). EM ELISAs were completed using anti-hIgG Fc capture antibody (109-005-098, Jackson Immunoresearch). Samples were detected using an anti-hIgG Fc F(ab′)2 (709-036-098, Jackson Immunoresearch) as previously published.^16^ To increase specificity for the samples obtained from cynomolgus monkeys, the EM ELISA was adjusted to utilize mouse anti-hIgG (Bio-Rad MCA5748G) as the coating antibody. Similarly, the TM ELISA was adjusted to utilize horseradish peroxidase (HRP)-conjugated mouse anti-hIgG (Bio-Rad MCA5748G). The HRP conjugation was done in-house utilizing the LYNX Rapid HRP Antibody Conjugation Kit (Bio-Rad LNK002P).

### Statistical analysis and data visualization

GraphPad Prism (v8.4.3–9.5.0) was used to visualize data and determine statistical significance when appropriate. Ordinary one-way ANOVA was used to compare multiple groups with Tukey’s multiple comparisons test evaluating the means between groups. When one-way ANOVA was inappropriate, Brown–Forsythe and Welch ANOVA tests and Dunnett’s T3 multiple comparison were used instead. Statistical significance has been indicated as *P < 0.05, **P < 0.01, ***P < 0.001 and ****P < 0.0001. GraphPad Prism’s non-linear regression analysis (Sigmoidal, 4PL analysis) was used to interpolate both DFC concentrations and cytokine levels.

## Results

### In vitro activity

The *in vitro* potency of CTC-177 was evaluated using a screening panel containing 12 clinical isolates and representative ATCC strains of *K. pneumoniae*, *A. baumannii*, *P. aeruginosa* and *E. coli* (KAPE) displaying various susceptibilities to colistin and carbapenems. As shown in Table 1, CTC-177 demonstrated robust antibacterial activity against the whole panel including colistin-susceptible and -resistant strains. Given the very large molecular weight difference between the conjugate molecule and colistin, MIC values are shown in both mg/L and µM scales to facilitate comparison. CTC-177 was highly active with MIC ranges of 0.11–1.80 µM for *K. pneumoniae* and 0.11–0.90 µM for *A. baumannii*, while colistin MICs spread in wide ranges for these two species. CTC-177 showed similar potency towards *P. aeruginosa* and *E. coli* strains compared with colistin (individual MICs are shown in Table S1).

**Table 1.** Geometric mean MICs of colistin and CTC-177 across KAPE screening panel

Species	Concentration (mg/L)		Concentration (µM)	
	Colistin	CTC-177	Colistin	CTC-177
*K. pneumoniae*	0.81 (0.13–64)	28.76 (8–128)	0.69 (0.11–55.39)	0.41 (0.11–1.8)
*A. baumannii*	6.08 (0.25–128)	19.03 (8–64)	5.27 (0.43–110.78)	0.27 (0.11–0.9)
*P. aeruginosa*	0.39 (0.13–1)	20.89 (16–32)	0.34 (0.11–0.87)	0.29 (0.23–0.45)
*E. coli*	0.20 (0.13–0.5)	20.89 (8–64)	0.17 (0.11–0.43)	0.29 (0.11–0.9)

CTC-177 was subjected to a 52-strain screening panel possessing various susceptibilities to colistin. Geometric mean MIC and (range) displayed for each species to colistin and CTC-177 in mg/L and µM concentrations.

### In vivo efficacy

The *in vivo* efficacy of CTC-177 was first evaluated in a deep-thigh infection mouse model as described previously.^24^ Mice were administered CTC-177 at 30 mg/kg via IP injection 12 h prior to infection and a booster dose of 30 mg/kg 1 h following infection with *A. baumannii* bacteria [colistin MIC 1 mg/L (0.87 µM), CTC-177 MIC 8 mg/L (0.11 µM)]. Control-group mice were treated with colistin or vehicle starting from 1 h post infection. CTC-177 immunoprophylaxis resulted in significantly lower thigh bacterial burdens than vehicle control and colistin treatment (Table 2). The strong immunoprophylactic effect of CTC-177 in the thigh model supported further evaluation of this compound in other clinically relevant *in vivo* models.

**Table 2.** Bacterial containment comparison in multiple models of infection

Infection	Organ	Burden Reduction (log_10_ cfu/organ or log_10_ cfu/mL of blood)	
		Colistin	CTC-177
*A. baumannii* deep tissue	Thigh	1.62	2.17
*A. baumannii* pneumonia	Lung	0.11	0.54
*A. baumannii* septicaemia	Lung	3.09	3.35
	Kidney	3.71	4.33
	Blood	1.86	3.06
*K. pneumoniae* septicaemia	Lung	3.03	4.03
	Kidney	3.69	4.20
	Blood	4.18	4.54
*P. aeruginosa* septicaemia	Lung	1.61	2.03
	Kidney	1.80	1.84
	Blood	1.30	1.60

Burden reduction (log_10_ cfu/organ or log_10_ cfu/mL of blood) compared with vehicle control at 48 h (*A. baumannii* and *K. pneumoniae*) or 24 h (*P. aeruginosa*) post infection. Colistin administered at 2.5 mg/kg, SC, twice daily except in the pneumonia model, where colistin was escalated to 10 mg/kg, SC, twice daily. CTC-177 was administered twice at 30 mg/kg at −12 h and +1 h relative to infection.

Considering pneumonia and septicaemia are of more clinical concern compared with soft-tissue infections,^4,9,17–21^ we established both neutropenic mouse models of septicaemia and pneumonia using strains of various Gram-negative species and assessed efficacy of CTC-177 in these models. In the pneumonia model due to colistin-sensitive *A. baumannii*, colistin treatment [5 mg/kg (not shown) or 10 mg/kg, subcutaneously (SC), twice daily] resulted in little to no reduction of bacterial burdens even when the suggested dosing level was exceeded,^25^ consistent with what others have observed.^26^ However, CTC-177 prophylaxis (30 mg/kg, IP, −12 h and +1 h relative to infection) led to a modest, but significant reduction compared with both vehicle and colistin treatment (Table 2). In the septicaemia model induced by the same *A. baumannii* strain, immunoprophylaxis with CTC-177 (30 mg/kg, IP, −12 h and +1 h) resulted in a 3.35 log bacterial burden reduction in the lungs and a 4.33 log reduction in the kidneys compared with vehicle control, similar to what was observed with colistin treatment (2.5 mg/kg, SC, twice daily), where 3.09 and 3.71 log reductions were observed in the lungs and kidneys, respectively (Table 2). Moreover, CTC-177 reduced blood burdens by 3.06 logs, significantly better than the 1.86 log reduction due to colistin treatment (Table 2). Similarly, CTC-177 prophylaxis was highly effective in suppressing bacterial burdens in septicaemia models due to colistin-sensitive *K. pneumoniae* [colistin MIC 0.25 mg/L (0.22 µM), CTC-177 MIC 16 mg/L (0.23 µM)] and *P. aeruginosa* [colistin MIC 0.25 mg/L (0.22 µM), CTC-177 MIC 32 mg/L )0.45 µM)] strains (Table 2). Of note, the *P. aeruginosa* septicaemia model was a 24 h model, in which survival of vehicle-treated mice was 20%, whereas CTC-177 prophylaxis improved survival to 80%.

It is worth noting that prophylaxis with either Fc alone or colistin had no effect on bacterial burdens (Figure S2a).

### Optimization of prophylactic regimen

To simplify the prophylactic regimen, we carried out studies using the *Acinetobacter* septicaemia model to assess efficacy as well as tolerance of a single dose of CTC-177 administered 12 h prior to infection. We found 5, 10, 20, 30 and 60 mg/kg CTC-177 to be well tolerated with dose-dependent efficacy up until 60 mg/kg (Figure 2). The 60 mg/kg group in all three target sites (lungs, kidneys and blood) and 30 mg/kg group in kidneys and blood demonstrated burden reduction similar to colistin, but the remaining lower-dose regimens of CTC-177 were not as effective as colistin treatment. In a separate study, we tested CTC-177 at 60 and 90 mg/kg 12 h prior to infection and observed comparable bacterial burdens, but increased weight loss in the 90 mg/kg group (not shown), indicating that 60 mg/kg was the most effective dose (data not shown). We next explored the optimal window to administer the single-dose prophylaxis of CTC-177. Briefly, 60 mg/kg of CTC-177 was administered once at 72, 48, 24 or 12 h prior to infection. All groups receiving CTC-177 prophylaxis or colistin treatment had significantly lower bacterial burdens than vehicle control animals in lungs, kidneys and blood at 48 h post infection. A time-dependent trend was observed for burden levels in all target sites, where the longer the prophylactic window was, the less the burden reduction that was obtained. The best prophylactic efficacy was achieved with the −12 h administration, outcompeting treatment effects of colistin therapy, even though not statistically significant in lungs and kidneys (Figure 3a–c). Nevertheless, significant efficacy was achieved with CTC-177 prophylaxis as far out as 72 h prior to infection.

Consistent with efficacy observations, all CTC-177-pretreated animals had higher average weight at the endpoint of the study compared with vehicle controls (Figure 3d), further proving the effectiveness of this approach. We next sought to examine the effect of prophylaxis on the host inflammatory response, using cytokine/chemokine biomarkers in which elevated levels are associated with infection severity and/or disease progression, such as IL-6,^27–30^ TNFα^28,31,32^ and MIP-1α.^33,34^ As expected, elevation of plasma IL-6 (uninfected <1.5 pg/mL) and TNFα (uninfected 2.5 ± 0.4 pg/mL) observed in vehicle control animals were significantly dampened in CTC-177- or colistin-treated mice (*P* < 0.0001) (Figure 3e, Table S2). Similarly, plasma concentration of the macrophage chemoattractant MIP-1α (uninfected 3.0 ± 1 pg/mL) was also reduced in all treated groups compared with vehicle control (Figure 3e). Similar findings were observed at the infected tissue sites (lungs and kidneys), where IL-6 and MIP-1α were reduced in all treatment groups, while TNFα concentrations were highly variable (not shown).

The efficacy of this optimized single-dose prophylaxis regimen was further validated in *Klebsiella* and *Pseudomonas* septicaemia models (Figure S3, Figure S4), and demonstrated its superiority over the initially established two-dose regimen (Table S3).

### Using CTC-177 prophylaxis to sensitize *in vivo* response to colistin treatment

**Figure 4.**
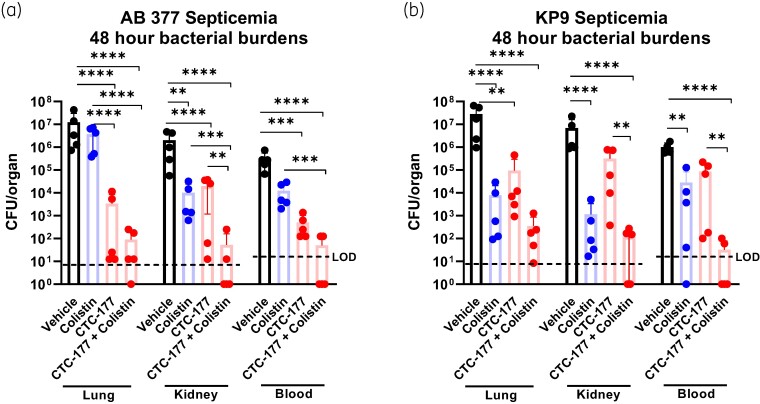
CTC-177 prophylaxis is effective against colistin-resistant infections. CTC-177 (60 mg/kg, IP) administered 12 h prior to infection. Colistin (2.5 mg/kg, SC) and vehicle administered twice daily commencing 1 h post infection with (a) colistin-resistant AB 377 or (b) colistin-sensitive KP9. Mice were sacrificed 48 h post infection, burdens displayed as the total burdens in the lung and kidney, or cfu/mL of blood at 48 h post infection. Dashed lines indicate LOD. The LOD was 8.3 cfu/organ (lung or kidney) and 20 cfu/mL of blood. Datapoints that fell below the LOD were estimated. **P < 0.01; ***P < 0.001; ****P < 0.0001 (*n* = 5). AB, *A. baumannii*; KP, *K. pneumoniae*.

To test whether the *in vitro* activity of CTC-177 against colistin resistance translates into *in vivo* efficacy, we used a colistin-resistant *A. baumannii* strain (AB 377, colistin MIC 8 mg/L) to establish the septicaemia mouse model. The *in vivo* response to colistin treatment was in line with the expectation in lungs, in which bacterial burdens were similar to that in untreated controls. However, there were moderate responses to colistin in kidneys and blood, regardless of the *in vitro* resistance phenotype of this strain, presumably due to higher concentrations of colistin in kidney tissue and blood compared with lung tissue. Nevertheless, single-dose prophylaxis of CTC-177 resulted in significantly lower burdens relative to vehicle control in the target sites (Figure 4a), with a few animals showing burden levels close to the limit of detection (LOD). Interestingly and encouragingly, CTC-177 prophylaxis reverted colistin resistance as animals that received colistin therapy following a single dose of CTC-177 prophylaxis demonstrated remarkably low burdens and even tissue sterilization, which was not observed in animals in prophylaxis-only or treatment-only groups. Compared with vehicle control, the average burden reduction due to the combination of CTC-177 prophylaxis and colistin treatment was 5.13, 4.58 and 3.58 log in lungs, kidneys and blood, respectively.

When the infecting organism was switched to *K. pneumoniae*, we again observed a similar synergistic effect of CTC-177 prophylaxis followed by colistin treatment, resulting in profound bacterial burden reduction and tissue sterilization (Figure 4b). Together, these findings illustrate a potential clinical application of CTC-177 as a highly effective adjuvant to colistin therapy, even in the case of colistin-resistant infections.

### PK properties

**Figure 5.**
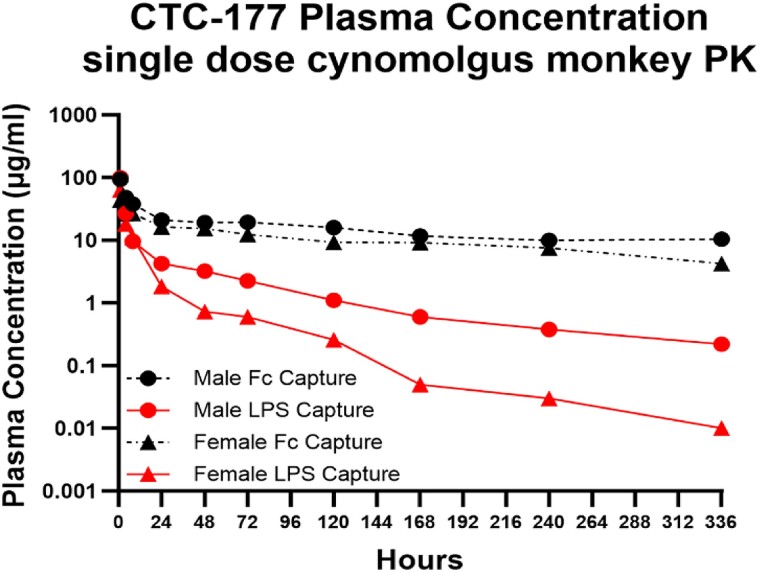
Single-dose cynomolgus monkey PK. A single IV bolus of CTC-177 was administered at 22 mg/kg in one female and one male cynomolgus monkey. Blood was collected at 1, 4, 8, 24, 48, 72, 120, 168, 240 and 336 h post dose. Plasma concentrations at each timepoint were measured by sandwich ELISA. The lower LOD of PK ELISA was 0.45 ng/mL.

PK properties of CTC-177 were assessed in mice and cynomolgus monkeys. In mice, following a single IV bolus dose of CTC-177 at 20 mg/kg, a terminal half-life of 24.1 and 24.3 h and an AUC_0–24_ of 214.1 and 212.6 μg/mL·h was found in males and females, respectively (Table S4, Figure S5). We then evaluated PK in cynomolgus monkeys, where one male and one female monkey were administered a single IV bolus (∼3 min) of CTC-177 at 22 mg/kg. Blood was collected through to 336 h post dose. We found that after an initial rapid TM degradation in the first 24 h (∼20% and ∼11% intact ADC in male and female, respectively, at 24 h post dose), CTC-177 demonstrated a long terminal half-life (115 h in male and 65 h in female) (Figure 5, Table 3). This is highly encouraging as it suggests that CTC-177 is likely to have a stronger protective effect in humans than what was observed in mice due to allometric scaling differences in the PK in mice versus cynomolgus monkeys.

**Table 3.** Summary of PK parameters for CTC-177 in cynomolgus monkeys by non-compartmental analysis

Sex	ELISA	*t*_½_ (h)	*C*_max_ (μg/mL)	AUC_0–t_ (μg/mL·h)	AUC_0–inf_ (μg/mL·h)	CL (mL/h/kg)	4 h TM/EM (%)	24 h TM/EM (%)
M	LPS-capture	115.95	99.01	839.16	875.85	25.12	55.6	20.1
	Fc-capture	265.74	93.49	5176.46	9177.86	2.40		
F	LPS-capture	65.69	63.73	444.50	445.37	49.40	68.9	11.2
	Fc-capture	171.83	44.01	3450.05	4503.14	4.89		

Plasma CTC-177 concentrations of whole compound (LPS-capture) and degraded compound (Fc-capture) were calculated utilizing ELISA.

## Discussion

Antibiotic resistance causes a significant reduction in antibiotic options for clinicians,^11,12^ resulting in prolonged treatment and extended hospital stays, which potentiate antibiotic resistance and spread of infection. Many groups are working to modify and improve existing therapeutics to slow resistance.^35^ Others focus on new strategies to combat antibiotic resistance such as the use of antibodies due to their stability and prolonged circulation.

Currently, three antibodies are approved to treat bacterial infections caused by *Clostridioides difficile* and *Bacillus anthracis*, with many more antibacterial antibodies in development, as reviewed elsewhere.^36^ Some of the antibodies in development are antibody–antibiotic conjugates (AACs, alternatively ADCs or DFCs), which not only have the benefit of extended circulation, but which also deliver a potent antibiotic.^36,37^ Unfortunately, many of the antibodies and AACs in development are specific to an individual bacterial species.

The Cloudbreak™ platform utilizes and improves these strategies by conjugating novel polymyxin-derived dimers, which successfully target colistin-resistant Gram-negative bacteria via a non-cleavable linker to the Fc region of hIgG1.

In the studies presented here, we have demonstrated that CTC-177 prophylaxis effectively reduces bacterial burdens in mouse models of deep tissue infection, pneumonia and septicaemia, all of which are clinical concerns for Gram-negative pathogens.^4,9,17–21^ Single-dose prophylaxis of CTC-177 was efficacious in limiting bacterial burdens, comparable to or better than daily treatment of colistin. Moreover, effective prophylaxis can be achieved as far as 72 h prior to infection and as low as 10 mg/kg, which is a testament of the clinical potential for DFCs to combat bacterial infections.

Of note, the prophylactic efficacy of CTC-177 was achieved in both colistin-sensitive and -resistant infections. Most encouragingly, in a scenario of colistin-resistant *A. baumannii* infection, a single dose of CTC-177 at 60 mg/kg in mice was effective to restore the effect of colistin as a potent therapy, which otherwise would fail. The effect of such a combinatory regimen was highly effective as complete sterilization was achieved in 60% of the kidneys and blood and 20% of lungs.

We found that initiating CTC-177 treatment 1 h post infection reduced its efficacy (Figure S2b). This is likely due to the treatment initiation delay caused by the size of CTC-177 and its relatively slow absorption following IP administration (plasma *T*_max_ 2 h, not shown). The impact on morbidity and mortality due to delayed treatment, including colistin treatment, has been observed by others.^38–42^ This reinforces the benefit of utilizing CTC-177 as a prophylactic agent.

We are encouraged by the prolonged PK observed in cynomolgus monkeys, which suggests that CTC-177 may have a stronger pharmacodynamic effect in humans than in mice. We are still working to understand the remarkable drug exposure difference in male and female cynomolgus monkeys and to understand the mechanism of initial rapid TM degradation in the context of the observed efficacy even when administered 72 h prior to infection.

The mouse models presented here are not suitable for toxicity evaluation. However, the kidneys collected from animals treated with CTC-177 were generally much healthier than those treated with colistin or vehicle control. Furthermore, in the mouse PK study performed on healthy mice, we did not observe any visible kidney damage at 24 h post a single dose of CTC-177. Given these findings, we recently performed a pilot experiment in which CTC-177, dosed IV at 40 mg/kg, was well tolerated in both male and female Sprague–Dawley rats, with no adverse effects observed including clinical pathology, organ weights and microscopic findings (data not shown). These pilot studies support the further development of CTC-177.

In summary, we demonstrate that CTC-177 offers promise as a broad-spectrum prophylactic agent with two potential clinical applications. First, the extended half-life in cynomolgus monkeys and predicted pharmacodynamic effect in humans, taken together with the observed prophylactic efficacy of CTC-177, indicate that administering a DFC such as CTC-177 prior to elective procedures, intubation or IV catheterization would reduce the likelihood of bacterial infection. Second, the restored efficacy of colistin for treatment of colistin-resistant infections following CTC-177 prophylaxis has critical implications for clinical practice, as it may provide a valuable solution to the dilemma caused by MDR Gram-negative pathogens that are also resistant to colistin.

## Supplementary Material

dlae100_Supplementary_Data

## Supplementary data

Figures S1 to S5 and Tables S1 to S4 are available as Supplementary data at *JAC-AMR* Online.

## References

[1] Trubiano JA, Padiglione AA. Nosocomial infections in the intensive care unit. Anaesth Intensive Care Med 2015; 16: 598–602. 10.1016/j.mpaic.2015.09.010

[2] CDC . Antibiotic Resistance Threats in the United States, 2019. 2019. https://stacks.cdc.gov/view/cdc/82532.

[3] CDC . COVID-19: U.S. Impact on Antimicrobial Resistance, Special Report 2022. 2022. https://stacks.cdc.gov/view/cdc/119025.

[4] Eliopoulos GM, Maragakis LL, Perl TM. *Acinetobacter baumannii*: epidemiology, antimicrobial resistance, and treatment options. Clin Infect Dis 2008; 46: 1254–63. 10.1086/529198 18444865

[5] Vuotto C, Longo F, Balice MP et al Antibiotic resistance related to biofilm formation in *Klebsiella pneumoniae*. Pathogens 2014; 3: 743–58. 10.3390/pathogens3030743 25438022 PMC4243439

[6] Skov RL, Monnet DL. Plasmid-mediated colistin resistance (*mcr-1* gene): three months later, the story unfolds. Euro Surveill 2016; 21: 30155. 10.2807/1560-7917.ES.2016.21.9.30155 26967914

[7] Moradali MF, Ghods S, Rehm BHA. *Pseudomonas aeruginosa* lifestyle: a paradigm for adaptation, survival, and persistence. Front Cell Infect Microbiol 2017; 7: 39. 10.3389/fcimb.2017.00039 28261568 PMC5310132

[8] Navon-Venezia S, Kondratyeva K, Carattoli A. *Klebsiella pneumoniae*: a major worldwide source and shuttle for antibiotic resistance. FEMS Microbiol Rev 2017; 41: 252–75. 10.1093/femsre/fux013 28521338

[9] Wong D, Nielsen TB, Bonomo RA et al Clinical and pathophysiological overview of *Acinetobacter* infections: a century of challenges. Clin Microbiol Rev 2017; 30: 409–47. 10.1128/CMR.00058-16 27974412 PMC5217799

[10] Pang Z, Raudonis R, Glick BR et al Antibiotic resistance in *Pseudomonas aeruginosa*: mechanisms and alternative therapeutic strategies. Biotechnol Adv 2019; 37: 177–92. 10.1016/j.biotechadv.2018.11.013 30500353

[11] Zilberberg MD, Shorr AF, Micek ST et al Multi-drug resistance, inappropriate initial antibiotic therapy and mortality in Gram-negative severe sepsis and septic shock: a retrospective cohort study. Crit Care 2014; 18: 596. 10.1186/s13054-014-0596-8 25412897 PMC4264255

[12] Bassetti M, Peghin M, Vena A et al Treatment of infections due to MDR Gram-negative bacteria. Front Med (Lausanne) 2019; 6: 74. 10.3389/fmed.2019.00074 31041313 PMC6477053

[13] Cai Y, Wang X, Zhang T et al Rational design of a potent antimicrobial peptide based on the active region of a gecko cathelicidin. ACS Infect Dis 2024; 10: 951–60. 10.1021/acsinfecdis.3c00575 38315114

[14] Chang RYK, Chow MYT, Wang Y et al The effects of different doses of inhaled bacteriophage therapy for *Pseudomonas aeruginosa* pulmonary infections in mice. Clin Microbiol Infect 2022; 28: 983–9. 10.1016/j.cmi.2022.01.006 35123053

[15] Fan X, Li X, Ren H et al *rgpA*-Engineered/functionalized DNA vaccine as a novel prophylactic vaccination to prevent *Porphyromonas gingivalis*-induced periodontitis: an *in vivo* study. Discov Med 2024; 36: 355 65. 10.24976/Discov.Med.202436181.33 38409840

[16] Lovey A, Krel M, Borchardt A et al Development of novel immunoprophylactic agents against multidrug-resistant Gram-negative bacterial infections. Antimicrob Agents Chemother 2021; 65: e0098521. 10.1128/AAC.00985-21 34370589 PMC8522721

[17] Joly-Guillou ML . Clinical impact and pathogenicity of *Acinetobacter*. Clin Microbiol Infect 2005; 11: 868–73. 10.1111/j.1469-0691.2005.01227.x 16216100

[18] Chung DR, Song J-H, Kim SH et al High prevalence of multidrug-resistant nonfermenters in hospital-acquired pneumonia in Asia. Am J Respir Crit Care Med 2011; 184: 1409–17. 10.1164/rccm.201102-0349OC 21920919

[19] Chopra T, Marchaim D, Awali RA et al Epidemiology of bloodstream infections caused by *Acinetobacter baumannii* and impact of drug resistance to both carbapenems and ampicillin-sulbactam on clinical outcomes. Antimicrob Agents Chemother 2013; 57: 6270–5. 10.1128/AAC.01520-13 24100492 PMC3837851

[20] Chopra T, Marchaim D, Johnson PC et al Risk factors and outcomes for patients with bloodstream infection due to *Acinetobacter baumannii-calcoaceticus* complex. Antimicrob Agents Chemother 2014; 58: 4630–5. 10.1128/AAC.02441-14 24890594 PMC4135982

[21] Freire MP, de Oliveira Garcia D, Garcia CP et al Bloodstream infection caused by extensively drug-resistant *Acinetobacter baumannii* in cancer patients: high mortality associated with delayed treatment rather than with the degree of neutropenia. Clin Microbiol Infect 2016; 22: 352–8. 10.1016/j.cmi.2015.12.010 26711434

[22] Rutishauser U, Cunningham BA, Bennett C et al Amino acid sequence of the Fc region of a human gamma G-immunoglobulin. Proc Natl Acad Sci U S A 1968; 61: 1414–21. 10.1073/pnas.61.4.1414 5249817 PMC225271

[23] CLSI . Methods for Dilution Antimicrobial Susceptibility Tests for Bacteria That Grow Aerobically-Tenth Edition: M07. 2015.

[24] Craig WA, Redington J, Ebert SC. Pharmacodynamics of amikacin *in vitro* and in mouse thigh and lung infections. J Antimicrob Chemother 1991; 27 Suppl C: 29–40. 10.1093/jac/27.suppl_C.29 1830302

[25] Tsuji BT, Pogue JM, Zavascki AP et al International consensus guidelines for the optimal use of the polymyxins: endorsed by the American College of Clinical Pharmacy (ACCP), European Society of Clinical Microbiology and Infectious Diseases (ESCMID), Infectious Diseases Society of America (IDSA), International Society for Anti-infective Pharmacology (ISAP), Society of Critical Care Medicine (SCCM), and Society of Infectious Diseases Pharmacists (SIDP). Pharmacotherapy 2019; 39: 10–39. 10.1002/phar.2209 30710469 PMC7437259

[26] Aoki N, Tateda K, Kikuchi Y et al Efficacy of colistin combination therapy in a mouse model of pneumonia caused by multidrug-resistant *Pseudomonas aeruginosa*. J Antimicrob Chemother 2009; 63: 534–42. 10.1093/jac/dkn530 19147523

[27] Hack CE, de Groot ER, Felt-Bersma RJ et al Increased plasma levels of interleukin-6 in sepsis. Blood 1989; 74: 1704–10. 10.1182/blood.V74.5.1704.1704 2790194

[28] Sullivan JS, Kilpatrick L, Costarino AT Jr et al Correlation of plasma cytokine elevations with mortality rate in children with sepsis. J Pediatr 1992; 120: 510–5. 10.1016/S0022-3476(05)82476-X 1552388

[29] Song J, Park DW, Moon S et al Diagnostic and prognostic value of interleukin-6, pentraxin 3, and procalcitonin levels among sepsis and septic shock patients: a prospective controlled study according to the Sepsis-3 definitions. BMC Infect Dis 2019; 19: 968. 10.1186/s12879-019-4618-7 31718563 PMC6852730

[30] Greenhill CJ, Rose-John S, Lissilaa R et al IL-6 trans-signaling modulates TLR4-dependent inflammatory responses via STAT3. J Immunol 2011; 186: 1199–208. 10.4049/jimmunol.1002971 21148800

[31] Glauser MP, Zanetti G, Baumgartner J-D et al Septic shock: pathogenesis. Lancet 1991; 338: 732–6. 10.1016/0140-6736(91)91452-Z 1679876

[32] Gharamti AA, Samara O, Monzon A et al Proinflammatory cytokines levels in sepsis and healthy volunteers, and tumor necrosis factor-alpha associated sepsis mortality: a systematic review and meta-analysis. Cytokine 2022; 158: 156006. 10.1016/j.cyto.2022.156006 36044827

[33] DiPietro LA, Burdick M, Low QE et al MIP-1alpha as a critical macrophage chemoattractant in murine wound repair. J Clin Invest 1998; 101: 1693–8. 10.1172/JCI1020 9541500 PMC508751

[34] de Pablo R, Monserrat J, Prieto A et al Role of circulating soluble chemokines in septic shock. Med Intensiva 2013; 37: 510–8. 10.1016/j.medin.2012.09.008 23158870

[35] Laws M, Shaaban A, Rahman KM. Antibiotic resistance breakers: current approaches and future directions. FEMS Microbiol Rev 2019; 43: 490–516. 10.1093/femsre/fuz014 31150547 PMC6736374

[36] Cavaco M, Castanho MARB, Neves V. The use of antibody-antibiotic conjugates to fight bacterial infections. Front Microbiol 2022; 13: 835677. 10.3389/fmicb.2022.835677 35330773 PMC8940529

[37] Mariathasan S, Tan M-W. Antibody–antibiotic conjugates: a novel therapeutic platform against bacterial infections. Trends Mol Med 2017; 23: 135–49. 10.1016/j.molmed.2016.12.008 28126271

[38] Zasowski EJ, Bassetti M, Blasi F et al A systematic review of the effect of delayed appropriate antibiotic treatment on the outcomes of patients with severe bacterial infections. Chest 2020; 158: 929–38. 10.1016/j.chest.2020.03.087 32446623

[39] Samrah S, Bashtawi Y, Hayajneh W et al Impact of colistin-initiation delay on mortality of ventilator-associated pneumonia caused by *A. baumannii*. J Infect Dev Ctries 2016; 10: 1129–34. 10.3855/jidc.7203 27801377

[40] Chastre J, Fagon J-Y. Ventilator-associated pneumonia. Am J Respir Crit Care Med 2002; 165: 867–903. 10.1164/ajrccm.165.7.2105078 11934711

[41] Le Moyec L, Racine S, Le Toumelin P et al Aminoglycoside and glycopeptide renal toxicity in intensive care patients studied by proton magnetic resonance spectroscopy of urine. Crit Care Med 2002; 30: 1242–5. 10.1097/00003246-200206000-00013 12072675

[42] Tigen ET, Koltka EN, Dogru A et al Impact of the initiation time of colistin treatment for *Acinetobacter* infections. J Infect Chemother 2013; 19: 703–8. 10.1007/s10156-013-0549-1 23393014

