# Supplementary material for: CTC-177, a novel drug–Fc conjugate, shows promise as an immunoprophylactic agent against multidrug-resistant Gram-negative bacterial infections
Source: JAC Antimicrob Resist. 2024 Jul 26;6(4):dlae100. doi: 10.1093/jacamr/dlae100 (PMC11276960; doi:10.1093/jacamr/dlae100)
Supplement: dlae100_Supplementary_Data [file dlae100_supplementary_data.docx]

**CTC-177, a novel drug-Fc conjugate, shows promise as an immunoprophylactic agent against multidrug resistant Gram-negative bacterial infections.**

Arianne LOVEY^1^, Annie LEE^1^, Allison YU^1^, Mila KREL^1^, Mingming WANG^1^, Padmaja PADERU^1^, Thomas BRADY^2^, Grayson HOUGH^2^, Qiping ZHAO^2^, James M. BALKOVEC^2^, David S. PERLIN^1,3,4^*, and Yanan ZHAO^1,3^

^1^ Center for Discovery and Innovation, Hackensack Meridian Health, Nutley, NJ 07110

^2^ Cidara Therapeutics, Inc, San Diego, CA 92121

^3^ Hackensack Meridian School of Medicine, Nutley, NJ 07110

^4^ Georgetown University Lombardi Comprehensive Cancer Center, Washington, DC 20057

* Address correspondence to David S. Perlin, Ph.D. [david.perlin@hmh-cdi.org](mailto:david.perlin@hmh-cdi.org), phone: 201-880-3500

**Supplementary Tables and Figures**

| Table S1: MIC comparison of colistin and CTC-177 | | | | |
| --- | --- | --- | --- | --- |
| Strain | Concentration (mg/L) | | Concentration (µM) | |
| Internal ID | Colistin | CTC-177 | Colistin | CTC-177 |
| KP 1 | 64 | 128 | 55.39 | 1.80 |
| KP 2 | 0.25 | 16 | 0.22 | 0.23 |
| KP 3 | 4 | 32 | 3.46 | 0.45 |
| KP 4 | 0.25 | 16 | 0.22 | 0.23 |
| KP 5 | 2 | 128 | 1.73 | 1.80 |
| KP 6 | 0.25 | 16 | 0.22 | 0.23 |
| KP 7 | 0.25 | 32 | 0.22 | 0.45 |
| KP 8 | 0.125 | 8 | 0.11 | 0.11 |
| KP 9 | 0.25 | 16 | 0.22 | 0.23 |
| KP 10 | 0.25 | 16 | 0.22 | 0.23 |
| KP 11 | 0.25 | 8 | 0.22 | 0.11 |
| KP 12 | 64 | 128 | 55.39 | 1.80 |
| KP ATCC (A. 700603) | 0.25 | 64 | 0.22 | 0.90 |
| AB 1 | 32 | 8 | 27.70 | 0.11 |
| AB 2 | 16 | 32 | 13.85 | 0.45 |
| AB 3 | 16 | 8 | 13.85 | 0.11 |
| AB 4 | 8 | 16 | 6.92 | 0.23 |
| AB 5 | 8 | 16 | 6.92 | 0.23 |
| AB 6 (AB 377) | 8 | 16 | 6.92 | 0.23 |
| AB 7 | 128 | 32 | 110.78 | 0.45 |
| AB 8 | 0.3 | 32 | 0.26 | 0.45 |
| AB 9 | 1 | 64 | 0.87 | 0.90 |
| AB 10 | 0.5 | 16 | 0.43 | 0.23 |
| AB 11 | 32 | 32 | 27.70 | 0.45 |
| AB 12 (AB 482) | 1 | 8 | 0.87 | 0.11 |
| PA 1 | 0.125 | 16 | 0.11 | 0.23 |
| PA 2 | 2 | 16 | 1.73 | 0.23 |
| PA 3 | 0.25 | 16 | 0.22 | 0.23 |
| PA 4 | 0.25 | 16 | 0.22 | 0.23 |
| PA 5 | 1 | 32 | 0.87 | 0.45 |
| PA 6 | 0.5 | 32 | 0.43 | 0.45 |
| PA 7 | 0.5 | 16 | 0.43 | 0.23 |
| PA 8 | 0.25 | 32 | 0.22 | 0.45 |
| PA 9 | 1 | 32 | 0.87 | 0.45 |
| PA 10 | 0.25 | 16 | 0.22 | 0.23 |
| PA 11 | 0.5 | 16 | 0.43 | 0.23 |
| PA 12 | 0.25 | 16 | 0.22 | 0.23 |
| PA01 (A. 10145) | 0.25 | 32 | 0.22 | 0.45 |
| PA ATCC (A. 27853) | 0.25 | 16 | 0.22 | 0.23 |
| EC 1 | 0.25 | 32 | 0.22 | 0.45 |
| EC 2 | 0.125 | 32 | 0.11 | 0.45 |
| EC 3 | 0.5 | 16 | 0.43 | 0.23 |
| EC 4 | 0.25 | 16 | 0.22 | 0.23 |
| EC 5 | 0.125 | 16 | 0.11 | 0.23 |
| EC 6 | 0.25 | 32 | 0.22 | 0.45 |
| EC 7 | 0.25 | 32 | 0.22 | 0.45 |
| EC 8 | 0.125 | 16 | 0.11 | 0.23 |
| EC 9 | 0.125 | 16 | 0.11 | 0.23 |
| EC 10 | 0.25 | 64 | 0.22 | 0.90 |
| EC 11 | 0.25 | 16 | 0.22 | 0.23 |
| EC 12 | 0.125 | 8 | 0.11 | 0.11 |
| EC ATCC (A. 25922) | 0.25 | 16 | 0.22 | 0.23 |

MICs of each individual strain to colistin and CTC-177 in mg/L and µM concentrations.

| Table S2: Inflammatory levels in uninfected animals | | | | | | |
| --- | --- | --- | --- | --- | --- | --- |
| Cytokine/ Chemokine | Plasma (pg/mL) | | Lung (pg/mL) | | Kidney (pg/mL) | |
|  | Average | SD | Average | SD | Average | SD |
| IL-6 | <1.50 | NA | 1.78 | 0.39 | 109.79 | 20.34 |
| TNFα | 2.45 | 0.44 | 17.60 | 4.43 | 733.60 | 90.25 |
| MIP1α | 2.96 | 1.04 | 31.87 | 8.76 | 133.11 | 8.04 |

Undiluted sample used to calculate background levels of IL-6, TNFα and MIP1α in uninfected animals. Plasma IL-6 was below the limit of detection for all samples. N=5.

| Table S3: Single prophylactic 60mg/kg dose of CTC-177 improves efficacy over previously used -12h+1h dosing scheme | | | | |
| --- | --- | --- | --- | --- |
| Infection | Organ | Log Burden Reduction | | |
|  |  | Colistin 2.5 mg/kg BID | CTC-177 30 mg/kg -12+1 | CTC-177 60 mg/kg -12 |
| *A. baumannii* Septicemia | Lung | 3.09 | 3.35 | 4.34 |
|  | Kidney | 3.71 | 4.33 | 4.36 |
|  | Blood | 1.86 | 3.06 | 4.68 |
| *K. pneumoniae* Septicemia | Lung | 2.09 | 2.07 | 2.27 |
|  | Kidney | 2.39 | 1.77 | 1.99 |
|  | Blood | 1.58 | 1.27 | 1.54 |
| *P. aeruginosa* Septicemia | Lung | 1.59 | 2.19 | 3.03 |
|  | Kidney | 0.76 | 1.36 | 1.67 |
|  | Blood | 1.22 | 0.75 | 1.21 |

CTC-177 administered at 30mg/kg, IP, -12h/+1h or 60mg/kg, IP, -12h. Colistin administered at 2.5mg/kg, SC, BID. Log burden reduction compared to vehicle control at 48h (*A. baumannii* and *K. pneumoniae*) or 24h (*P. aeruginosa*) post infection.

| Table S4: CTC-177 murine PK | | | | | | |
| --- | --- | --- | --- | --- | --- | --- |
| Sex | ELISA | T1/2 (h) | Cmax (μg/ml) | AUC0-24 (μg/ml*h) | 4h TM/EM (%) | 24h TM/EM (%) |
| M | LPS-capture | 24.08 | 32.07 | 214.10 | 6.19 | 3.87 |
|  | Fc-capture | 75.25 | 174.23 | 3612.00 |  |  |
| F | LPS-capture | 24.27 | 17.23 | 212.60 | 8.51 | 8.73 |
|  | Fc-capture | 66.89 | 122.76 | 2293.00 |  |  |

The PK parameters for CTC-177 were evaluated in male and female CD-1 mice (4-5 animals/group) after 20 mg/kg IV administration. Whole blood samples were collected via tail vein at 0.3, 1, 2, 4, 8, 24, or cardiac puncture at 48 hours post injection. Plasma DFC concentrations of whole compound (LPS) and degraded compound (Fc) were calculated utilizing ELISA. PK parameters calculated utilizing non-compartmental analysis.

**Figure S1: Synthesis of the small molecule TM coupling partner of CTC-177**

**Step a. Preparation of dimethyl 2,2'-(((3S,4S)-pyrrolidine-3,4-dicarbonyl)bis(azanediyl))(2S,2'S,3S,3'S)-bis(3-methylpentanoate)**

EDC (3.2 g, 17 mmol) was added to a stirring mixture of racemic-trans1-(*tert*-butoxycarbonyl)pyrrolidine-3,4-dicarboxylic acid **Compound A** (2 g, 7.7 mmol), isoluecine methyl ester HCl salt (3.5 g, 19.3 mmol), HOBt (2.6 g, 17 mmol), and triethylamine (2 g, 20 mmol) in 15 mL of DMF. The reaction was stirred for 12 hours, diluted with aqueous 1N HCl (100 mL), extracted into ethyl acetate, dried over sodium sulfate and concentrated. The diastereomers were separated by reversed phase liquid chromatography (RPLC) using an Isco Combiflash liquid chromatograph eluted with 15% to 95% acetonitrile and water using 0.1 % TFA modifier. The more polar isomer was pooled and lyophilized to afford 1.2 g of the Boc-protected ester. Yield, combined 59%.LCMS [M-(1boc)+H^+^) = 414.2. The polar isomer Boc-protected ester (1.2 g, 2.3 mmol)) was stirred in 30 mL of 4M HCl (g) in dioxane for 30 minutes. The solvent was removed by rotary evaporation and dried under high vacuum to afford **Compound B** as an HCl salt as a white solid. Yield of 0.60 g, 75%. LCMS [M+H^+^) = 414.2.

**Step b. Preparation of (2S,2'S,3S,3'S)-2,2'-(((3S,4S)-1-(4,7,10,13-tetraoxahexadec-15-ynoyl)pyrrolidine-3,4-dicarbonyl)bis(azanediyl))bis(3-methylpentanoic acid)**

HATU (389 mg, 0.87 mmol) was added to a stirring mixture of the dimethyl 2,2'-(((3S,4S)-pyrrolidine-3,4-dicarbonyl)bis(azanediyl))(2S,2'S,3S,3'S)-bis(3-methylpentanoate) HCl salt (325 mg, 0.72 mmol), the propargyl-peg4 acid (226 mg, 0.86 mmol), and triethylamine (291 mg. 2.9 mmol) and stirred at ambient temperature for 30 minutes. The mixture was purified directly by reversed phase liquid chromatography (RPLC) using an Isco Combiflash liquid chromatograph eluted with 20% to 95% acetonitrile and water using 0.1 % TFA modifier. Fractions showing desired ions by LCMS (M+H+) = 656.4 were pooled and concentrated. The residue was stirred in a 1:1:2 mixture of methanol/THF/DI water containing LiOH (52 mg, 2.2 mmol) at ambient temperature for 30 minutes. The reaction mixture was acidified with a few drops of acetic acid and the volume was reduced by half on the rotary evaporator. The mixture was purified by reversed phase liquid chromatography (RPLC) using an Isco Combiflash liquid chromatograph eluted with 10% to 95% acetonitrile and water using 0.1 % TFA modifier. The pure fractions were pooled and lyophilized to afford **Compound C** as a clear oil. Yield of 375 mg, 83%, 2 steps. Positive ions were found by LCMS [M+H+) = 626.2.

**Step c. Preparation of di-tert-butyl (((2S,5R,8S,11S,14S,17S,22S)-22-(2-aminoacetamido)-5-benzyl-11-(2-((tert-butoxycarbonyl)amino)ethyl)-17-((R)-1-hydroxyethyl)-8-isobutyl-3,6,9,12,15,18,23-heptaoxo-1,4,7,10,13,16,19-heptaazacyclotricosane-2,14-diyl)bis(ethane-2,1-diyl))dicarbamate**

To a mixture of tri-Boc-protected PMB heptapeptide **Compound D** (4.24 g, 4 mmol) (1) and Z-Gly-OH (1 g, 4.8 mmol) in anhydrous DMF (8 mL) was added HATU (1.87 g, 4.9 mmol) in portions over 20 minutes, followed by DIPEA (936 mg, 7.2 mmol). After the reaction mixture was stirred for 15 minutes, it was poured into water (100 mL). The white solid product was collected by filtration and washed with water. The material was re-dissolved in MeOH (50 mL) and treated with Pd/C (5%) (1 g), then stirred under a hydrogen atmosphere (balloon pressure) overnight. The Pd/C (5%) was then filtered, concentrated and the residue purified by RPLC (150 g, 15 to 75 % MeOH and water). Yield of **Compound E** was 4.02 g, 90%. Ions found by LCMS: [(M – Boc + 2H)/2]^+^ = 510.4, [(M – 3Boc + 2H)/2]^+^ = 410.2

**Step d. Preparation of di-tert-butyl (((2S,5R,8S,11S,14S,17S,22S)-22-(2-((2S,3R)-2-amino-3-hydroxybutanamido)acetamido)-5-benzyl-11-(2-((tert-butoxycarbonyl)amino)ethyl)-17-((R)-1-hydroxyethyl)-8-isobutyl-3,6,9,12,15,18,23-heptaoxo-1,4,7,10,13,16,19-heptaazacyclotricosane-2,14-diyl)bis(ethane-2,1-diyl))dicarbamate**

To a mixture of di-tert-butyl (((2S,5R,8S,11S,14S,17S,22S)-22-(2-aminoacetamido)-5-benzyl-11-(2-((tert-butoxycarbonyl)amino)ethyl)-17-((R)-1-hydroxyethyl)-8-isobutyl-3,6,9,12,15,18,23-heptaoxo-1,4,7,10,13,16,19-heptaazacyclotricosane-2,14-diyl)bis(ethane-2,1-diyl))dicarbamate (4.02 g, 3.592 mmol) and Z-Thr-OH (980.3 mg, 3.87 mmol) in anhydrous DMF (5 mL) was added HATU (1.47 g, 3.87 mmol) in portions over 10 minutes, followed by DIPEA (755 mg, 5.8 mmol). After the addition, the reaction was stirred for 20 minutes and then poured into water (100 mL). The white solid product was collected by filtration and washed with water. The material was re-dissolved in MeOH (50 mL) and treated with Pd/C (5%) (1 g), and stirred under hydrogen overnight. Pd/C was then filtered, and the filtrate was concentrated and purified by RPLC (150 g column, 15 to 80 % MeOH and water). Yield of **Compound F** was 3.68 g, 84%. Ion found by LCMS: [(M - 2Boc + 2H)/2]^+^ = 511.0

**Step e. Preparation of tert-butyl (2-((2S,5R,8S,11S,14S,17S,22S)-22-((8S,11S)-8-amino-11-((R)-1-hydroxyethyl)-2,2-dimethyl-4,9,12-trioxo-3-oxa-5,10,13-triazapentadecan-15-amido)-5-benzyl-11,14-bis(2-((tert-butoxycarbonyl)amino)ethyl)-17-((R)-1-hydroxyethyl)-8-isobutyl-3,6,9,12,15,18,23-heptaoxo-1,4,7,10,13,16,19-heptaazacyclotricosan-2-yl)ethyl)carbamate (S)-2-(((benzyloxy)carbonyl)amino)-4-((tert-butoxycarbonyl)amino)butanoate**

A mixture of di-tert-butyl (((2S,5R,8S,11S,14S,17S,22S)-22-(2-((2S,3R)-2-amino-3-hydroxybutanamido)acetamido)-5-benzyl-11-(2-((tert-butoxycarbonyl)amino)ethyl)-17-((R)-1-hydroxyethyl)-8-isobutyl-3,6,9,12,15,18,23-heptaoxo-1,4,7,10,13,16,19-heptaazacyclotricosane-2,14-diyl)bis(ethane-2,1-diyl))dicarbamate (1.2 g, 0.984 mmol) and Z-Dab(Boc)-OH DCHA salt (605 mg, 1.13 mmol) was dissolved in anhydrous NMP (3 mL). It was treated with HATU (430 mg, 1.13 mmol) in portions over 5 minutes, followed by DIPEA (150 mg, 1.13 mmol). The reaction was stirred for 30 minutes and then directly purified by RPLC (100 g column, 40 to 100 % MeOH and water). The collected fractions were concentrated by rotary evaporation to a white solid (Ion found by LCMS: [M - 2Boc + 2H)/2]^+^ = 678). The material was re-dissolved in MeOH (30 mL) and treated with Pd/C (5%), then stirred under a hydrogen atmosphere (balloon pressure) overnight. The Pd/C was filtered, and the filtrate was concentrated by rotary evaporation and further dried under high vacuum. Yield of **Compound G** was 1.07 g, 76.6 %. Ion found by LCMS: [(M - 2Boc + 2H)/2]^+^ = 611.

**Step f. Preparation of (3S,4S)-N3,N4-bis((2S,3S)-1-(((S)-4-amino-1-(((2S,3R)-3-hydroxy-1-oxo-1-((2-oxo-2-(((3S,6S,9S,12S,15R,18S,21S)-6,9,18-tris(2-aminoethyl)-15-benzyl-3-((R)-1-hydroxyethyl)-12-isobutyl-2,5,8,11,14,17,20-heptaoxo-1,4,7,10,13,16,19-heptaazacyclotricosan-21-yl)amino)ethyl)amino)butan-2-yl)amino)-1-oxobutan-2-yl)amino)-3-methyl-1-oxopentan-2-yl)-1-(4,7,10,13-tetraoxahexadec-15-ynoyl)pyrrolidine-3,4-dicarboxamide**

The title compound was prepared by coupling tert-butyl (2-((2S,5R,8S,11S,14S,17S,22S)-22-((8S,11S)-8-amino-11-((R)-1-hydroxyethyl)-2,2-dimethyl-4,9,12-trioxo-3-oxa-5,10,13-triazapentadecan-15-amido)-5-benzyl-11,14-bis(2-((tert-butoxycarbonyl)amino)ethyl)-17-((R)-1-hydroxyethyl)-8-isobutyl-3,6,9,12,15,18,23-heptaoxo-1,4,7,10,13,16,19-heptaazacyclotricosan-2-yl)ethyl)carbamate (S)-2-(((benzyloxy)carbonyl)amino)-4-((tert-butoxycarbonyl)amino)butanoate **Compound G** (2.0 eq) and (2S,2'S,3S,3'S)-2,2'-(((3S,4S)-1-(4,7,10,13-tetraoxahexadec-15-ynoyl)pyrrolidine-3,4-dicarbonyl)bis(azanediyl))bis(3-methylpentanoic acid) **Compound C** (1.0 eq) using EDC (2.1 eq) and HOBt (2.2 eq) in DMF at room temperature for 12 hours. The product was purified directly by (RPLC) using an Isco Combiflash liquid chromatograph eluted with 30% to 100% methanol and water using 0.1 % TFA modifier. The fractions containing the desired product (LCMS analysis) were pooled and concentrated to give **Compound H**. The Boc-protected dimer was stirred in a 1/1 mixture of TFA/DCM (10 mL) at ambient temperature for 30 minutes. The solvent was removed by rotary evaporation and dried under high vacuum. The crude product was purified by reversed phase liquid chromatography (RPLC) using an Isco Combiflash liquid chromatograph eluted with 0% to 90% acetonitrile and water using 0.1 % TFA modifier. The pure fractions were pooled and lyophilized to afford **Compound I** as the octa-TFA salt. The yield of the desired product was 54% for the 2 steps. Positive mass ions were found by LCMS (M+3H^+^)/3 = 877.8, LCMS (M+4H^+^)/4 = 658.8, LCMS (M+5H^+^)/5 = 527.4, and LCMS (M+6H^+^)/6 = 439.8.

**Reference**

**1** Magee TV, Brown MF, Starr JT, Ackley DC, Abramite JA, Aubrecht J, Butler A, Crandon JL, Dib-Hajj F, Flanagan ME, Granskog K, Hardink JR, Huband MD, Irvine R, Kuhn M, Leach KL, Li B, Lin J, Luke DR, MacVane SH, Miller AA, McCurdy S, McKim JM, Jr., Nicolau DP, Nguyen TT, Noe MC, O'Donnell JP, Seibel SB, Shen Y, Stepan AF, Tomaras AP, Wilga PC, Zhang L, Xu J, Chen JM. 2013. Discovery of Dap-3 polymyxin analogues for the treatment of multidrug-resistant Gram-negative nosocomial infections. Journal of medicinal chemistry 56:5079-5093.


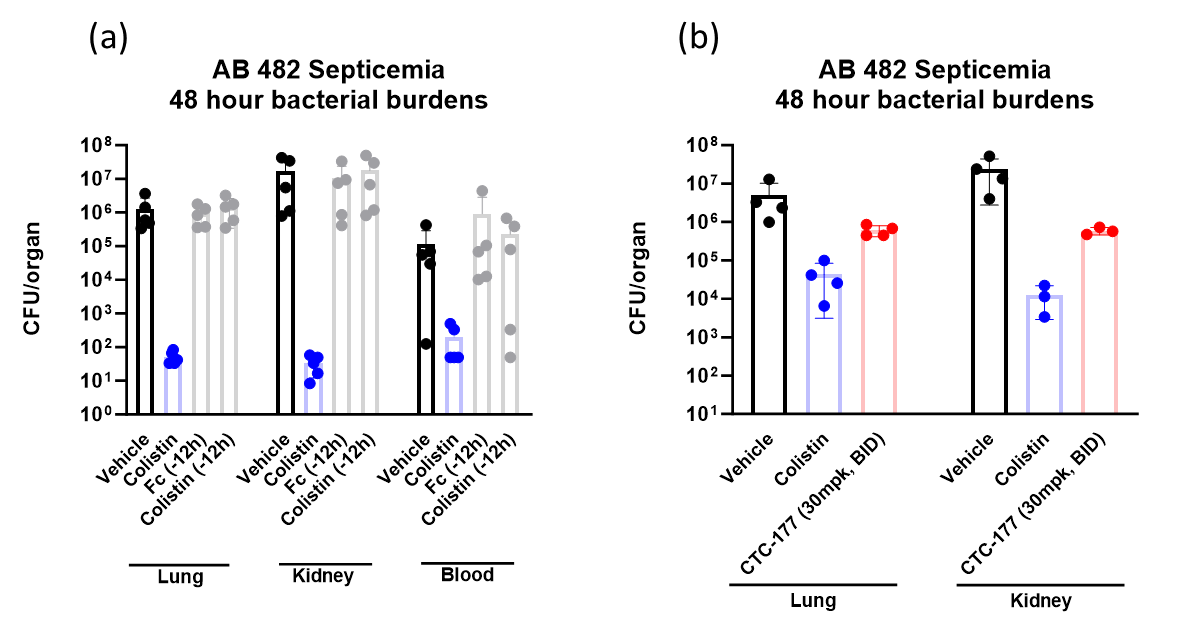
**Figure S2: Prophylaxis using Fc portion of CTC-177 or colistin has no effect.** (A) Fc fragment (60 mg/kg, IP) or colistin (2.5mg/kg, IP) administered at 12h prior infection with *A. baumannii* as indicated. Colistin (2.5 mg/kg, SC, BID) or vehicle control (PBS, IP, BID) were initiated at 1h post infection. (B) CTC-177 (30 mg/kg, IP, BID), Colistin (2.5 mg/kg, SC, BID) or vehicle control (PBS, IP, BID) initiated at 1h post *A. baumannii* infection. (A-B) Mice were sacrificed 48h post infection, burdens displayed as the total burdens in the lung and kidney, or CFU/mL of blood at 48h post infection. (A) N=5, (B) N=4.


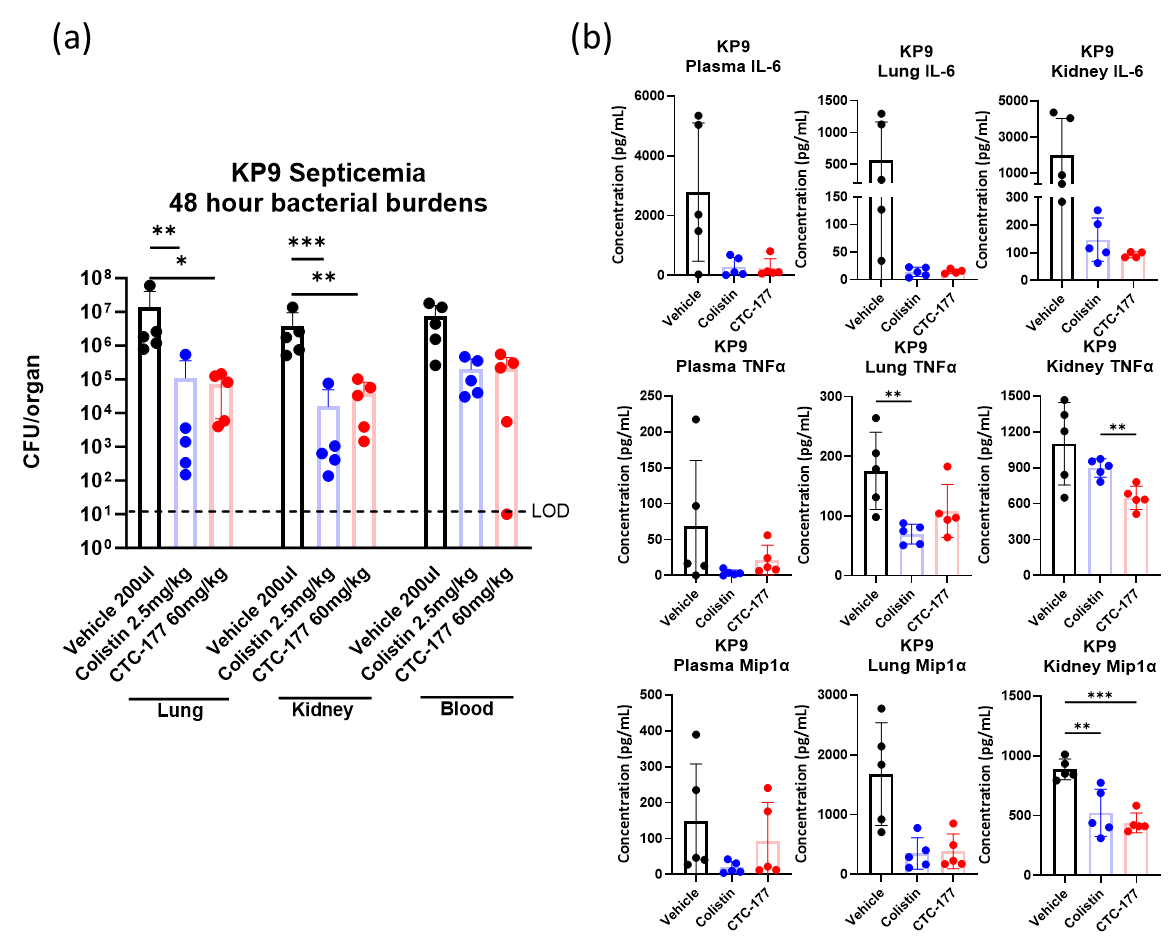


**Figure S3: CTC-177 60mg/kg improves *K. pneumoniae* outcomes.** Single prophylactic dose of CTC-177 administered IP at 60mg/kg 12h prior to infection with K. pneumoniae. Colistin (2.5mg/kg, SC) and vehicle administered BID commencing 1h post infection. Mice were sacrificed 48h post infection, (A) burdens displayed as the total burdens in the lung and kidney, or CFU/mL of blood at 48h post infection. (B) IL-6, TNFα and MIP1α levels in plasma, lung lysate and kidney lysate at 48h post infection. *, P<0.05; **, P< 0.01; ***, P<0.001; ****, P<0.0001. N=5.

**
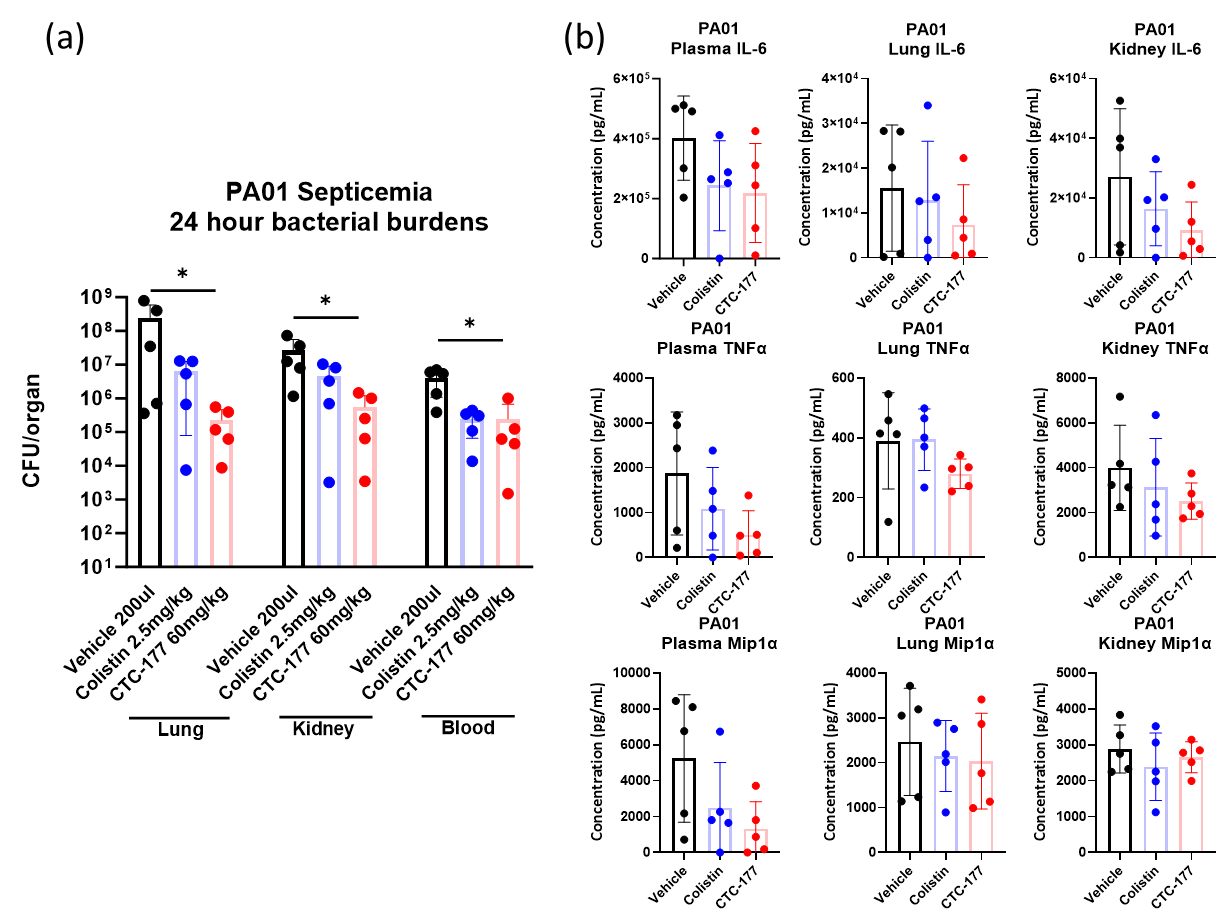
**

**Figure S4: CTC-177 60mg/kg improves *P. aeruginosa* outcomes.** Single prophylactic dose of CTC-177 administered IP at 60mg/kg 12h prior to infection with P. aeruginosa. Colistin (2.5mg/kg, SC) and vehicle administered BID commencing 1h post infection. Mice were sacrificed 24h post infection, (A) burdens displayed as the total burdens in the lung and kidney, or CFU/mL of blood at 24h post infection. (B) IL-6, TNFα and MIP1α levels in plasma, lung lysate and kidney lysate at 24h post infection. *, P<0.05; **, P< 0.01; ***, P<0.001; ****, P<0.0001. N=5.

**Figure S5: Time-concentration curve of CTC-177 after IV administration of a single dose of CTC-177 at 20 mg/kg in mice (n=4 or 5 per time point).**
